# Heme Oxygenase-1 as a Novel Metabolic Player

**DOI:** 10.1155/2013/814058

**Published:** 2013-12-05

**Authors:** Hun-Taeg Chung, Stefan W. Ryter, Hong Pyo Kim

**Affiliations:** ^1^School of Biological Sciences, University of Ulsan, Ulsan 680-749, Republic of Korea; ^2^Department of Pulmonary and Critical Care Medicine, Brigham and Women's Hospital, Harvard Medical School, Boston, MA 02115, USA; ^3^School of Pharmacy, Ajou University, Suwon 443-749, Republic of Korea

Heme oxygenase (HO; EC 1:14.99.3, heme, hydrogen donor: oxygen oxidoreductase (*α*-methene hydroxylating, decyclizing)) catalyzes the rate limiting the step in the oxidative catabolism of heme to generate biliverdin-IX*α*, which is subsequently converted to bilirubin-IX*α* by cytosolic NAD(P)H-dependent biliverdin reductase [1]. This reaction, which requires molecular oxygen as well as electrons from NADPH-dependent cytochrome p-450 reductase, liberates the small gas mediator carbon monoxide (CO) and ferrous iron [1]. The constitutive form of this enzyme (HO-2) is expressed highly in neuronal and vascular tissues, whereas the inducible form of this enzyme (HO-1) is recognized as a major stress-inducible protein in mammalian cells [2]. HO-1 induction represents a general inducible response in cells and tissues by a broad range of chemical and physical stress agents [3], and it is transcriptionally regulated in response to these agents primarily by the Keap1–Nrf2 system, a master regulator of the oxidative stress response [4].

The inducible form of HO, heme oxygenase-1 (HO-1), confers protection against oxidative stress conditions *in vitro* and *in vivo *[5]. Although the mechanisms of HO-1-dependent cytoprotection remain incompletely understood, accumulating evidence has implicated contributory roles for the products generated from HO activity. Biliverdin and bilirubin are potent antioxidant compounds, whereas iron liberated from HO activity stimulates the production of ferritin, a cytoprotective molecule [6]. Previously regarded as metabolic waste, CO may affect intracellular signaling pathways [7]. Exogenously applied CO can mimic the cytoprotective effects of HO-1 [8], involving the modulation of cellular redox state as well as the regulation of apoptosis, inflammation, and cellular proliferation [9]. 

In recent years, it has become clear that the HO-1/CO system can potentially impact cellular metabolic pathways. The cardinal example is the clearance of intracellular heme by the catabolic activity of HO-1 [1], leading to the redistribution of cellular iron [6, 10]. In this special issue, we sought to invite papers that explore the novel aspects of the HO-1/CO system with respect to the regulation of metabolic pathways. 

It is increasingly recognized that the mitochondria, the central energy generating organelle of the cell, can play important roles not only in metabolism but also in the regulation of cellular programs, including apoptosis and inflammation, and that mitochondrial dysfunction may be a key component of human diseases [11]. Recent studies have implicated HO-1/CO as important regulators of mitochondrial biogenesis and mitochondrial function [12, 13]. The paper by N. Rayamajhi et al., published in this special issue, demonstrates that the natural antioxidant quercetin, a plant derived flavonoid, enhances mitochondrial biogenesis through the activation of the HO-1/CO system in hepatocytes and furthermore prevents the decline of mitochondrial biogenesis in an *in vivo* model of sepsis. These studies lend support to the notion that natural dietary antioxidants, such as quercetin, could be used as pharmacological inducers of HO-1 and for the preservation of mitochondrial function for the treatment of disease. 

The role of the HO-1/CO system in the regulation of lipid metabolism is not well studied. The paper by S.-J. Lee et al. demonstrates that mitochondrial dysfunction under cellular stress conditions is associated with the disruption of lipid metabolism and the formation of lipid droplets (LD). Using HO-1 deficient mice, the authors uncover a previously unknown role for HO-1 in LD formation during polymicrobial sepsis. These results strongly suggest that HO-1 also influences lipid metabolism in response to mitochondrial dysfunction and as a part of the cytoprotective response to stress. 

In addition to lipids, polyamines are ubiquitous cellular constituents that can influence cell survival and death pathways. The relationship between the HO-1/CO system and polyamine metabolism is largely uncharted. The paper by H. Yang et al. demonstrates that spermidine, a cellular polyamine, can induce HO-1 in endothelial cells, through the Nrf2 pathway. The induction of HO-1 provides cellular antiapoptotic protection against the toxic effects of exogenous spermidine. These results affirm the antiapoptotic potential of HO-1 against natural apoptosis-inducing compounds and uncover a novel effect of polyamines on HO-1 regulation. 

It is generally recognized that HO-1/CO can provide protection in animal models of ischemia reperfusion injury and cardiovascular disease. In humans, susceptibility to ischemic disease is greater in males than in females. The paper by A. Pósa et al. explores the possibility that differences in HO-1 expression may account for gender differences in susceptibility to ischemic disease. The authors report that the differential HO activity may be responsible for the resistance of female versus male mice to cardiovascular disruption and vasoconstriction during ischemia. 

The therapeutic potential of CO has gained momentum in recent years and is summarized in the article by M. Knauert et al. The authors describe the intracellular signaling pathways that can be modulated by CO including mitogen activated protein kinases, nuclear factor *κ*-B, and the phosphatidylinositol-3-kinase/Akt pathway, which are involved in the regulation of inflammation and cell survival. Furthermore, the authors discuss the evolution of the field from animal modeling, in which CO has demonstrated tissue protective effects in lung injury and sepsis models, to its prospective use in the clinical arena as a therapy for human diseases. Although the latter goal is not yet realized, current efforts aim to characterize the efficacy of CO therapy for human sepsis. 

We hope that the articles presented in this special issue, representing current advances in the HO-1/CO field, with respect to their potential impact in metabolic pathways, will stimulate further exploration of this exciting area.

*Hun-Taeg Chung* *Hun-Taeg Chung* *Stefan W. Ryter* *Stefan W. Ryter* *Hong Pyo Kim* *Hong Pyo Kim*
